# Mild Ozone-Induced Oxidative Stress Modulates the Activity and Viability of Porcine Neutrophils and Monocytes

**DOI:** 10.3390/ani16020193

**Published:** 2026-01-08

**Authors:** Dominika Nguyen Ngoc, Jose Luis Valverde Piedra, Andrzej Milczak, Tomasz Szponder, Beata Drzewiecka, Aleksandra Pyzerska, Małgorzata Kowalczyk, Mateusz Fila, Ewa Tomaszewska, Arti Ahluwalia, Joanna Wessely-Szponder

**Affiliations:** 1 Sub-Department of Pathophysiology, Department of Preclinical Veterinary Sciences, Faculty of Veterinary Medicine, University of Life Sciences, Akademicka 12, 20-033 Lublin, Poland; dominika.nguyen@up.edu.pl (D.N.N.);; 2 Department of Pharmacology, Toxicology and Environmental Protection, Faculty of Veterinary Medicine, University of Life Sciences, Akademicka 12, 20-033 Lublin, Poland; jose.valverde@up.edu.pl; 3 Clinical Skills Laboratory, Faculty of Veterinary Medicine, University of Life Sciences, Głęboka 30, 20-612 Lublin, Poland; 4 Department of Diagnostics and Clinical Sciences, Faculty of Veterinary Medicine, University of Agriculture in Cracow, 31-120 Cracow, Poland; 5 Students Research Group of Veterinary Analysts, Sub-Department of Pathophysiology, Department of Preclinical Veterinary Sciences, Faculty of Veterinary Medicine, University of Life Sciences, Akademicka 12, 20-033 Lublin, Poland; 6 Department of Animal Physiology, Faculty of Veterinary Medicine, University of Life Sciences, Akademicka 12, 20-033 Lublin, Poland; ewa.tomaszewska@up.edu.pl; 7 Research Center “E. Piaggio” and Department of Information Engineering, University of Pisa, Largo Lucio Lazzarino 1, 56122 Pisa, Italy

**Keywords:** medical ozone treatment, oxidative stress, immunomodulation, neutrophils, monocytes, comparative medicine, porcine model, One Health

## Abstract

Ozone (O_3_) is a reactive oxidant increasingly applied in biomedical settings, yet its dose-dependent effects on innate immune cells, particularly those from non-human species, remain insufficiently defined. Within a One Health framework, this study examined how two clinically relevant O_3_ exposure regimens (30 µg/mL and 90 µg/mL) affect porcine neutrophils and monocytes isolated from peripheral blood. Cell viability, reactive oxygen and nitrogen species (RONS) production, and the activity of key enzymes (myeloperoxidase, elastase, alkaline phosphatase, arginase) were assessed at 1 h and 24 h post-exposure. The lower dose induced mild functional activation without compromising viability, whereas the higher dose triggered pronounced oxidative stress, enhanced degranulation, and reduced neutrophil viability by more than 60%. Neutrophils exhibited a stronger and more dynamic response than monocytes, which retained viability and differentiation capacity at 30 µg/mL but showed impaired function at 90 µg/mL. These findings highlight the dual nature of O_3_, where controlled exposure may support immunomodulation, while excessive dosing disrupts cell function. Defining safe and effective therapeutic windows remains critical for future applications.

## Simple Summary

Ozone is a reactive gas capable of modifying immune cell behaviour and is widely used in human and veterinary medicine. In this study, we examined how two ozone exposure regimens affected porcine neutrophils and monocytes obtained from peripheral blood. A lower dose (30 µg/mL for 10 min) induced mild oxidative activation without compromising cell viability, while a higher dose (90 µg/mL for 30 min) caused marked oxidative stress, increased release of inflammatory enzymes, and substantial loss of neutrophil viability. Monocytes were more resistant to oxidative injury, although they also showed impaired function at the higher dose. These findings demonstrate that ozone evokes dose-dependent and cell-type-specific responses, ranging from moderate immune modulation to cytotoxic effects. Because ozone is both an environmental pollutant and a medical agent, understanding its biological impact in animal models strengthens the One Health perspective, which recognises the interconnectedness of human, animal, and environmental health. Our results emphasise that careful control of ozone dose and exposure time is essential for ensuring safety and achieving the desired therapeutic effects across different species.

## 1. Introduction

Ozone (O_3_) is a triatomic allotrope of oxygen, generated and decomposed continuously: in the stratosphere it forms a UV-filtering layer through the reversible combination of atomic and molecular oxygen, while in the troposphere it arises as a photochemical byproduct of nitrogen oxides and sunlight. Despite its classification as an air pollutant at ground level, medical applications exploit ozone’s capacity to dissociate rapidly into reactive oxygen species (ROS), thereby enhancing local oxygen availability and ATP production. O_3_ initiates a cascade of secondary messengers that significantly influence and reorganise cellular redox signalling networks [1,2,3,4,5].

In blood, O_3_ dissolves in the aqueous phase and induces a mild oxidative stress by reacting with water and polyunsaturated fatty acids to generate hydrogen peroxide (H_2_O_2_) and a complex mixture of lipid ozonation products (LOPs), including lipoperoxyl radicals, hydroperoxides, malondialdehyde, isoprostanes, ozonides, alkenals, and 4-hydroxynonenal (4-HNE). These reactive species trigger endogenous protective mechanisms: transmembrane oxygen transport is enhanced, erythrocyte glycolysis accelerates, raising 2,3-bisphosphoglycerate levels and promoting oxygen release to tissues, the Krebs cycle and ATP production are stimulated, NADH levels drop while cytochrome C oxidation increases, and the synthesis of antioxidant enzymes (glutathione peroxidase, catalase, superoxide dismutase) and prostacyclin a potent vasodilator is induced [6,7,8,9,10].

The half-life of O_3_ in aqueous solutions is highly dependent on ambient temperature, which significantly affects its stability and reactivity in clinical applications. As temperature increases, O_3_ decomposes more rapidly due to enhanced molecular motion and acceleration of its reactions with dissolved organic and inorganic compounds. At 25 °C, the half-life of O_3_ is approximately 15 min while at 35 °C it is around 8 min [11,12,13,14]. Clinical use of O_3_ treatment back over 150 years: it was first employed as a disinfectant in 1856 and soon after to sterilize operating theaters and water supplies [15]. Today, medical O_3_ treatment is recognized for its antimicrobial, antioxidant, anti-inflammatory and immunomodulatory effects, with minimal reported side effects [1,3,16].

O_3_ treatment has been applied to a wide range of conditions, from chronic wounds and musculoskeletal disorders to infectious diseases. Widely used clinical applications in humans, its use is now being extended to animal health and veterinary practice. At low concentrations, O_3_ is believed to harness a hormetic effect, as sub-toxic doses trigger adaptive antioxidant, anti-inflammatory, and tissue-repair pathways without causing oxidative damage [17,18,19,20]. Given its promising safety profile, there is growing interest in adopting One Health principles to extend O_3_ treatment to veterinary practice, particularly in reproductive health across mammalian species. Future research must define species-specific dosing, delivery methods, and formulation stability to translate human protocols into safe and effective treatments for animals [4,21,22].

In human medicine, depending on the clinical objective, O_3_ can be administered systemically or locally, using a variety of established techniques [1,23]. Among systemic approaches, major blood ozonation (MBO) remains the most widely established technique. It involves the withdrawal of a defined volume of autologous blood, which is then exposed ex vivo to a precisely calibrated O_2_–O_3_ gas mixture, and subsequently reinfused into the patient [19,24,25]. Rectal insufflation is another well-documented and widely used method of O_3_ administration. Although inhalation of O_3_ is contraindicated due to its pulmonary toxicity, various alternative administration routes have been explored and validated, including topical, intramuscular, intradiscal, mucosal, and transdermal applications, as well as the use of ozonated water, oils, and gas mixtures [19,23,26,27,28,29].

Topical O_3_ applications utilize gas mixtures, ozonated water, or ozonated oils for the treatment of skin, mucosal, and cavity infections. Cutaneous lesions such as ulcers, burns, and wounds are treated using sealed ozone-resistant systems with humidified gas or compresses soaked in ozonated water, followed by overnight oil application. These methods are safe, cost-effective, and accelerate healing through disinfection, vasodilation, and improved tissue oxygenation [20,30,31,32].

In veterinary practice, the O_2_/O_3_ mixture is applied both systemically (MBO, intraperitoneal or rectal insufflation) and topically (ozonated oils, creams, foams) across a wide range of species [23,33]. Thanks to its antimicrobial, immunomodulatory and wound-healing properties, O_3_ treatment is used to treat chronic non-healing wounds, uterine infections, mucosal inflammations and other conditions in animals [19,21,34,35,36].

As already mentioned, medical ozone treatment does not cause major side effects. However, despite its proven benefits, concerns have been raised regarding its potential toxicity and the importance of adhering to strict administration protocols to ensure safety [18]. A concentration of 30 µg/mL ozone is frequently cited in the literature and used in MBO and local treatments, both in human clinical practice and in preclinical studies involving pigs. At this dose, O_3_ provides therapeutic effects, including analgesic, immunomodulatory and antimicrobial activity [37,38,39,40,41,42]. In contrast, concentrations around 90 µg/mL exceed the routinely recommended the therapeutic window for medical ozone. At this level, early hemolysis has been observed in vitro (approximately 4–5%), leading many authors to classify it as potentially toxic. Nevertheless, some in vivo studies suggest that certain tissues can tolerate such doses. In pigs, a single subarachnoid injection of 10 mL of an O_2_/O_3_ mixture at 90 µg/mL caused no changes in cerebrospinal fluid chemistry, nor did it result in abnormalities on MRI, CT imaging, or in spinal histopathology. Likewise, repeated rectal insufflations of ozone at the same concentration during organogenesis in Wistar rats did not produce any maternal toxicity, embryotoxicity, or teratogenic effects [43,44,45,46]. In efforts to better define the therapeutic window for medical ozone, previous studies have examined its effects on various blood cell types. While most investigations have focused on erythrocytes, some attention has also been given to leukocyte trafficking [25,47]. However, the impact of ozonation on the secretory activity and reactive oxygen species (ROS) generation by circulating leukocytes remains insufficiently characterised [48]. Neutrophils and monocytes are central to innate immunity and orchestrate the early phases of the inflammatory response. Early studies indicated that ozone can modulate neutrophil oxidative burst, adhesion, and degranulation, as well as influence macrophage differentiation, but these reports are largely outdated [29,49].

In addition, medical ozone has been shown to modulate the Kelch-like ECH-associated protein 1/nuclear factor erythroid 2–related factor 2 (Keap1/Nrf2) signalling pathway, which is widely recognised as a master regulator of antioxidant defence. By inducing a controlled and transient oxidative stimulus, ozone promotes Nrf2 activation and the expression of cytoprotective enzymes such as heme oxygenase-1 (HO-1), thereby enhancing cellular resistance to oxidative stress and contributing to its immunomodulatory effects [50,51,52]. Despite these advances, contemporary research has increasingly focused on clinical applications, leaving a gap in the mechanistic understanding of how circulating leukocytes respond to ozone exposure.

Therefore, the present study aimed to evaluate the dose-dependent effects of ozone on porcine neutrophils and monocytes. By assessing viability, nitrite accumulation, enzymatic activity, and macrophage differentiation under two defined exposure regimens (30 µg/mL for 10 min and 90 µg/mL for 30 min), we sought to update and expand the mechanistic evidence for ozone’s immunomodulatory potential, bridging older studies with current translational applications relevant to both human and veterinary medicine.

## 2. Materials and Methods

### 2.1. In Vitro Exposure of Porcine Leukocytes to Ozone

Neutrophils and monocytes were isolated from porcine blood collected from four animals at slaughter, according to previously described methods [53,54,55]. Each leukocyte subpopulation was divided into four groups (20 mL of cell suspension per condition): untreated control (C1), air stream–exposed control for 10 min (C2), and two ozone-treated groups exposed for 10 min (O3-30) or 30 min (O3-90), which yielded final dissolved O_3_ concentrations of approximately 30 µg/mL and 90 µg/mL, respectively. All exposures were performed at room temperature (approximately 25 °C). Ozone was generated using a MALATEC (Legnica, Poland) O_3_ generator. For the air-exposed control, ambient air was de-livered through the same device without activating O_3_ production [56]. The O_2_/O_3_ gas mixture was introduced into the cell suspensions through standard yellow polypropylene pipette tips (200 µL, Eppendorf, Hamburg, Germany) connected to the generator outlet and submerged in the medium. The gas flow was adjusted to produce fine bubbles, ensuring efficient gas–liquid transfer while avoiding excessive mechanical stress. Immediately after exposure, the cell suspensions were transferred to 24-well plates at a density of 2 × 10^6^ cells/mL and incubated at 37 °C in a humidified atmosphere containing 5% CO_2_.

Short-term exposure of leukocyte suspensions at 25 °C is widely used in in vitro ozone-cell contact models and does not compromise cell viability when followed by incubation under physiological conditions (37 °C, 5% CO_2_) [57]. Because ozone is highly unstable in aqueous media, with a half-life of approximately 15 min at 25 °C, the nominal concentrations of 30 µg/mL and 90 µg/mL represent the dissolved O_3_ measured immediately after bubbling the gas mixture for 10 and 30 min, respectively. Consequently, these two regimens differ both in peak O_3_ concentration and in total exposure duration and therefore constitute distinct combined oxidative doses rather than purely independent concentration variables. This interdependence should be taken into account when interpreting the observed cellular responses (Supplementary Note S1).

### 2.2. Assessment of Neutrophil and Monocyte Responses Following Ozone Exposure

Neutrophils were maintained in phosphate-buffered saline (PBS; with calcium and magnesium, Sigma-Aldrich, St. Louis, MO, USA) [53,58], whereas monocytes were cultured in Dulbecco’s Modified Eagle Medium (DMEM; low glucose, Sigma-Aldrich, USA) supplemented with 10% fetal bovine serum (FBS; Adlab, Warsaw, Poland) [59,60,61].

At 1 h and 24 h post-treatment, neutrophil function was analysed by measuring nitrite concentration with the Griess assay, superoxide anion (O_2_•^−^) production using the nitroblue tetrazolium (NBT) reduction test, and the release of activation markers including myeloperoxidase (MPO), alkaline phosphatase (ALP), elastase, and arginase.

Nitric oxide (NO) generation was determined by nitrite measurement as a stable end-product in the culture medium. Briefly, 100 µL of culture supernatant was mixed with 100 µL of Griess reagent (0.1% N-[1-naphthyl]ethylenediamine dihydrochloride, 1% sulfanilamide, and 2.5% H_3_PO_4_), incubated for 10 min at room temperature, and absorbance was recorded. Concentrations were calculated from a sodium nitrite standard curve (1.25–80 µM NaNO_2_) [62,63]. Superoxide anion production was quantified colorimetrically at 545 nm after 10 min incubation with NBT (0.1 mg/mL in PBS; Sigma-Aldrich, Poznań, Poland). Superoxide generation was expressed as nanomoles produced during the incubation period, calculated using an extinction coefficient of 21.1 nmol [58,64,65].

Neutrophil degranulation was evaluated by assessing the activity of enzymes released from azurophilic granules (MPO, elastase) and specific granules (ALP) [53,55,63]. Elastase activity was determined spectrophotometrically using azocasein (Sigma-Aldrich) as substrate. Cell suspensions were incubated with the substrate at 25 °C for 10 min, and absorbance was measured at 490 nm with a BioTek EL800 microplate reader (BioTek, Janki, Poland). MPO release was quantified after incubation with o-phenylenediamine (Sigma-Aldrich, Poland), and ALP activity was determined under identical conditions using 4-nitrophenyl phosphate disodium salt hexahydrate (Sigma-Aldrich, Poland), with absorbance measured at 405 nm. Results for each enzyme were normalised to maximal degranulation (100% release) induced with 0.5% Triton X-100 (Sigma-Aldrich, Poland) and expressed as percentage values. Cytotoxicity was assessed using the MTT assay [63,66].

Arginase activity was determined by quantifying urea generated during arginase-mediated hydrolysis of L-arginine. After 24 h of culture, neutrophils were lysed with 50 μL of 0.1% Triton X-100 and incubated for 30 min. The lysates were supplemented with 50 μL of 25 mM Tris-HCl buffer and 10 μL of 10 mM MnCl_2_, and the enzyme was activated by heating at 55 °C for 10 min. Subsequently, 100 μL of 0.5 M L-arginine was added, and hydrolysis was carried out at 37 °C for 120 min. The reaction was stopped by adding 400 μL of an acid mixture (H_2_SO_4_/H_3_PO_4_/H_2_O, 1:3:7, *v*/*v*/*v*). Urea content was measured after addition of 40 μL of α-isonitrosopropiophenone (Sigma-Aldrich) and heating at 100 °C for 40 min. Urea concentrations were calculated using a standard curve ranging from 1 to 100 μg/mL [67,68].

Monocyte activity was analysed after 24 h of incubation. At this time, culture supernatants were collected for measurement of reactive oxygen and nitrogen species (RONS) [53,54]. Remaining adherent cells were provided with fresh medium and cultured for an additional 6 days under standard conditions (DMEM supplemented with 10% FBS) to allow spontaneous differentiation into macrophages. No exogenous cytokines or growth factors (e.g., M-CSF, GM-CSF) were applied. The medium was replaced every 2 days to maintain cell viability and differentiation [69,70].

### 2.3. Statistical Analysis

Statistical analysis was performed separately for neutrophil and monocyte assays. For neutrophils, data were analysed at the level of technical replicates. To compare treatment groups at each time point, we applied one-way ANOVA separately for 1 h and 24 h (groups: C1, C2, O3-30, O3-90), followed by Tukey’s post-hoc test. To examine within-group changes over time, we used paired *t*-tests on matched wells (1 h vs. 24 h). Results are reported as mean ± standard deviation (SD). Each condition comprised 16 wells per group, obtained as pooled means of duplicate readings from 8 plated wells. Assumptions were checked routinely, where homogeneity of variance was not met, we used Welch’s ANOVA with Games–Howell post hoc. Effect sizes for ANOVA are given as η^2^.

Monocyte assays were processed in the same way to ensure consistency of inference. Here, each condition included 6 wells per group, calculated as means of duplicate readings from 3 plated wells.

Statistical significance was assessed relative to the control groups: group a (untreated control—C1) and group b (air control—C2). Statistical significance was considered at (***: *p* ≤ 0.001; **: *p* ≤ 0.01; *: *p* ≤ 0.05). Results marked as n.s. indicate non-significant differences (*p* > 0.05). All analyses were performed using Statistica 13.3 (TIBCO, Palo Alto, CA, USA). Because animal identifiers were not available for these assays, statistics reflect technical-replicate variability rather than between-animal variability.

## 3. Results

### 3.1. Analysis of Ozone-Induced Viability, Enzymatic Activity and Morphological Alterations

#### 3.1.1. MTT Assay

**Figure 1:**
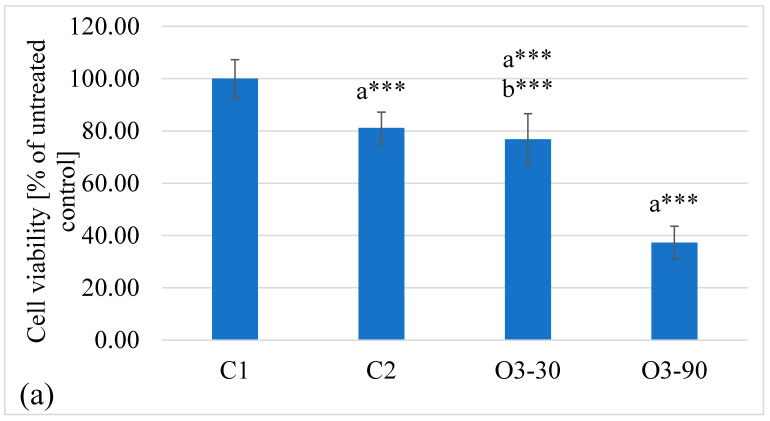

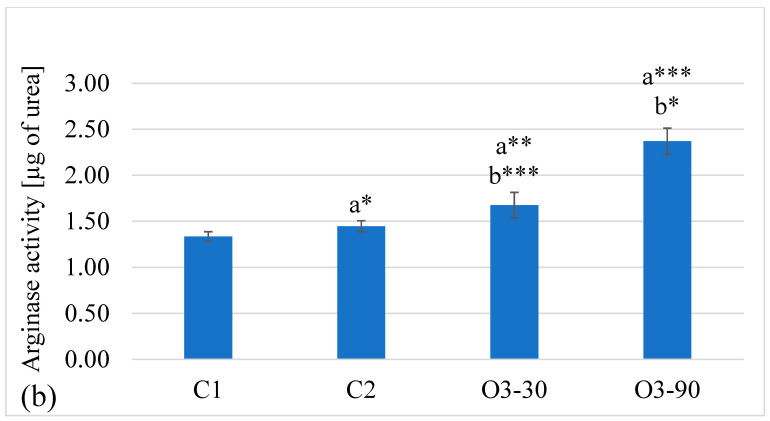
Effects of ozone exposure on neutrophil viability and arginase activity. Cell metabolic activity (**a**) and arginase activity (**b**) were measured after ozone treatment. Groups included: C1 (untreated control), C2 (air control), O3-30 (ozone, 30 µg/mL for 10 min), and O3-90 (ozone, 90 µg/mL for 30 min). Viability was assessed using the MTT assay and expressed as percentage of the untreated control (set as 100%), whereas arginase activity was quantified as µg of urea released after 24 h incubation. Data are shown as mean ± SD (n = 4 biological replicates, each measured in duplicate). Statistical analysis was performed using one-way ANOVA followed by Tukey’s multiple comparison test. Significant differences are indicated as * *p* ≤ 0.05, ** *p* ≤ 0.01, *** *p* ≤ 0.001. Letters indicate statistical comparison groups: a—versus C1, b—versus C2 (Tukey test).

Ozone exposure produced clear, dose-dependent effects on neutrophil metabolic activity. The high-dose group showed a marked reduction in viability compared with all other conditions, whereas moderate ozone induced only a minor decrease similar to the air control. Untreated controls displayed the highest metabolic activity, consistent with preserved cell viability. The full statistical outputs, including effect size and observed power, are summarised in Table 1. The corresponding data visualisation is presented in Figure 1a.

#### 3.1.2. Neutrophil Arginase Activity

Arginase activity exhibited a strong treatment-dependent response. High-dose ozone induced a pronounced increase relative to all other groups, while moderate ozone produced an intermediate elevation that exceeded both control conditions. Air exposure alone caused only a slight increase, indicating that the main effect was ozone-specific. Complete statistical results are provided in Table 1. The graphical representation of arginase activity is shown in Figure 1b.

#### 3.1.3. Myeloperoxidase Release

**Figure 2:**
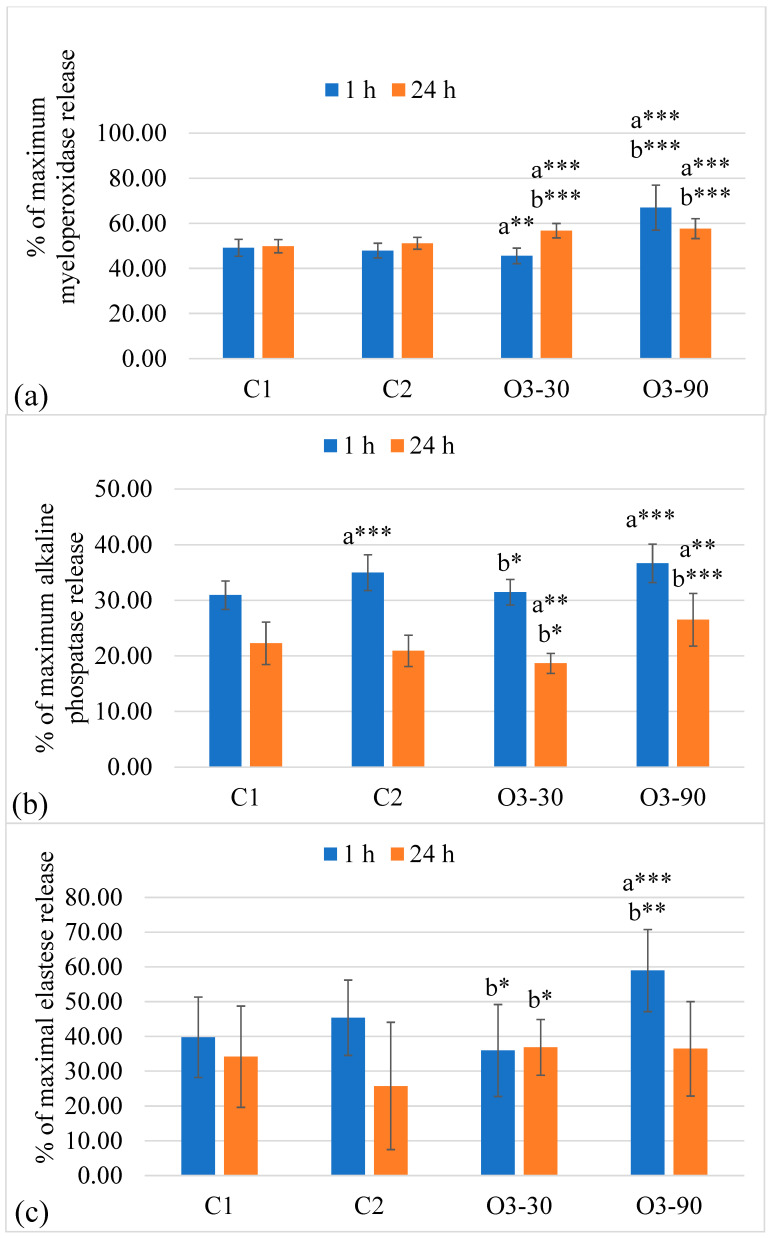
Neutrophil degranulation in response to ozone exposure. (**a**) Myeloperoxidase (MPO), (**b**) alkaline phosphatase (ALP), and (**c**) elastase release expressed as percentage of maximal enzyme release after 1 h and 24 h incubation. Bars represent mean ± SD (n = 4 biological replicates, each measured in duplicate). Statistical analysis was performed using two-way ANOVA with Tukey’s multiple comparison test. Significant differences are indicated as * *p* ≤ 0.05, ** *p* ≤ 0.01, *** *p* ≤ 0.001. Letters indicate statistical comparison groups: a—versus C1, b—versus C2 (Tukey test).

Ozone exposure induced clear dose- and time-dependent effects on MPO release. At 1 h, a strong treatment effect was driven by markedly elevated MPO levels in the high-ozone group compared with all other conditions. By 24 h, MPO release increased in both ozone-treated groups relative to the controls, although the difference between the two ozone doses was no longer significant. Temporal patterns varied by condition, with an early peak in the high-dose group that declined over time, while moderate ozone and the air control showed only modest increases. Full ANOVA statistics are presented in Table 2, and mean ± SD values with post hoc differences appear in Figure 2a.

#### 3.1.4. Alkaline Phosphatase Release

ALP release also showed a significant treatment effect at both time points. At 1 h, the high-ozone dose produced the greatest ALP release, whereas the moderate dose remained comparable to the untreated control. By 24 h, ALP levels decreased across most conditions, with the moderate-ozone group showing the lowest values and the high-dose group remaining elevated relative to the air control. Time-dependent suppression was evident in all groups except the high-ozone condition. ANOVA outcomes are summarised in Table 2, and detailed mean ± SD values with pairwise differences are shown in Figure 2b.

#### 3.1.5. Elastase Release

Elastase release exhibited a pronounced early peak in the high-ozone group, which exceeded all other conditions at 1 h. By 24 h, between-group differences were no longer significant, and overall elastase levels converged. A time-dependent decline was observed in the high-ozone and air-control groups, while the untreated and moderate-ozone conditions showed minimal temporal change. Complete statistical results are summarised in Table 2, and descriptive data with post hoc differences are presented in Figure 2c.

The combined pattern of reduced azurophilic and specific granule release together with enhanced arginase activity at 30 µg/mL further indicates a shift towards a regulated, immunomodulatory phenotype, in contrast to the clearly pro-inflammatory and cytotoxic profile induced by 90 µg/mL O_3_.

#### 3.1.6. Morphological Observation of Monocyte-Derived Macrophages Cell Viability

**Figure 3:**
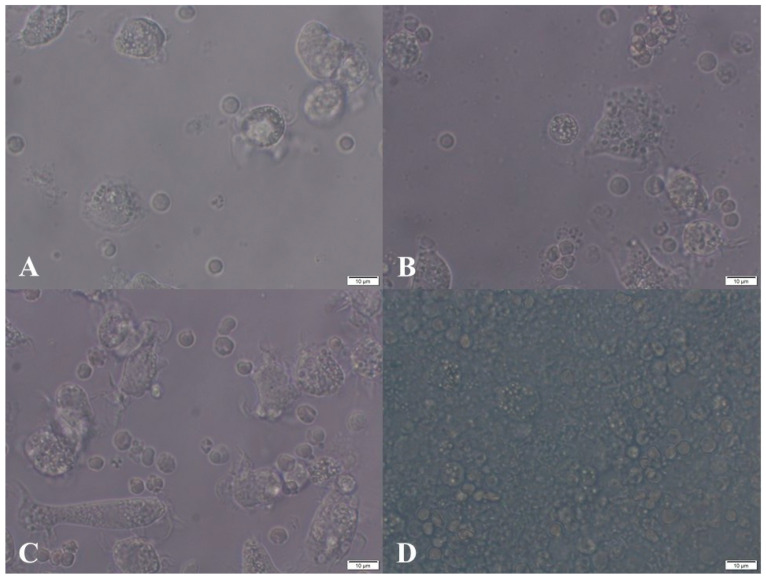
Phase-contrast microscopy of monocyte-derived macrophages on day 6 of culture following exposure to experimental conditions. Groups included: C1 (untreated control), C2 (air control), O3-30 (ozone, 30 µg/mL for 10 min), and O3-90 (ozone, 90 µg/mL for 30 min). Images represent (**A**) C1, (**B**) C2, (**C**) O3-30, and (**D**) O3-90. Cells were imaged after spontaneous differentiation under standard culture conditions. Scale bar: 10 µm.

Phase-contrast imaging was performed using an inverted microscope (Olympus CKX41, equipped with phase contrast optics and an XY stage). Morphological observation of monocyte-derived macrophages revealed a dose-dependent effect of O_3_ exposure on differentiation. Cells treated with O3-30 showed enhanced spreading and pseudopodia formation, characteristic of functional macrophages. In contrast, exposure to the higher dose of O3-90 impaired adhesion and resulted in rounded, densely clustered cells, indicating cytotoxic stress and suppressed maturation (Figure 3).

### 3.2. Detection of Reactive Oxygen and Nitrogen Species (RONS) in Culture Medium

#### 3.2.1. Neutrophil Response

**Figure 4:**
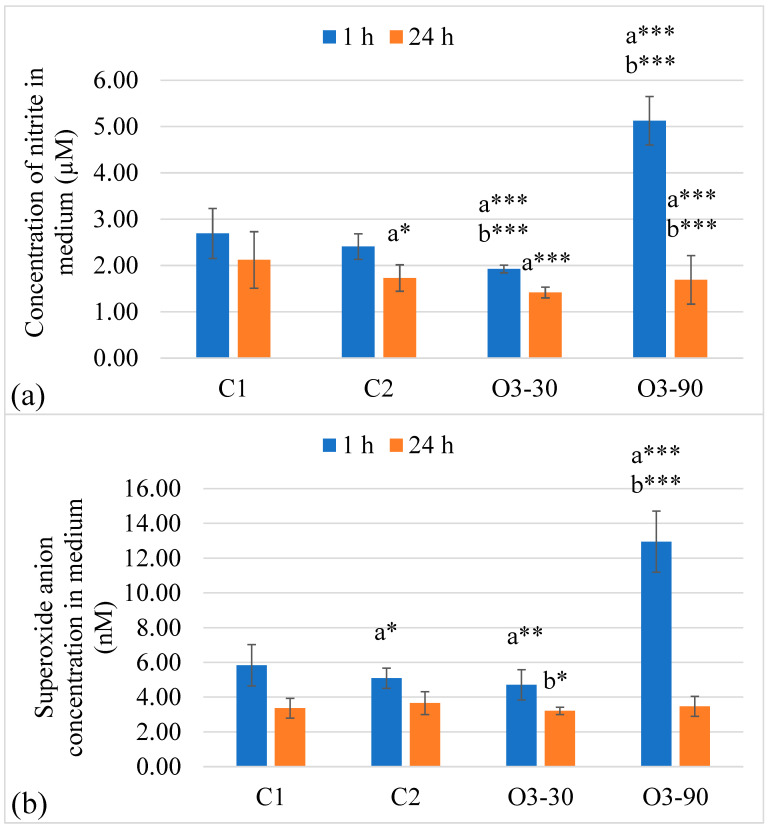
Nitrite (**a**) and superoxide (**b**) production in neutrophils following ozone exposure. (**a**) Nitrite concentrations measured by the Griess assay and (**b**) superoxide generation assessed by the NBT assay after 1 h and 24 h incubation. Bars represent mean ± SD (n = 4 biological replicates, each measured in duplicate). Statistical analysis was performed using two-way ANOVA with Tukey’s multiple comparison test. Significant differences are indicated as * *p* ≤ 0.05, ** *p* ≤ 0.01, *** *p* ≤ 0.001. Letters indicate statistical comparison groups: a—versus C1, b—versus C2 (Tukey test).

Ozone exposure induced clear time- and dose-dependent changes in nitrite accumulation. At 1 h, the high ozone dose produced the strongest increase, while the moderate dose yielded lower values than both controls. By 24 h, nitrite levels decreased across all conditions, and the overall treatment effect remained significant but less pronounced, with reductions mainly observed in the ozone-treated groups. All treatments showed significant declines between 1 h and 24 h. Mean ± SD values and post hoc differences are presented in Figure 4a, while the ANOVA model statistics are summarised in Table 3.

Superoxide generation demonstrated a similar pattern, with a strong treatment effect observed at 1 h. The high ozone dose resulted in markedly elevated superoxide levels, whereas the moderate dose showed a slight reduction relative to the untreated control. By 24 h, superoxide concentrations decreased in all groups, and the overall between-group effect was no longer significant. Detailed ANOVA parameters are presented in Table 3, while mean ± SD values with post hoc comparisons are shown in Figure 4b.

Taken together, the profile observed at 30 µg/mL, characterised by suppression of excessive RONS generation, is consistent with a controlled adaptive response rather than overt activation, supporting a potential immunomodulatory effect of mild O_3_ exposure.

#### 3.2.2. Monocyte Response

**Figure 5:**
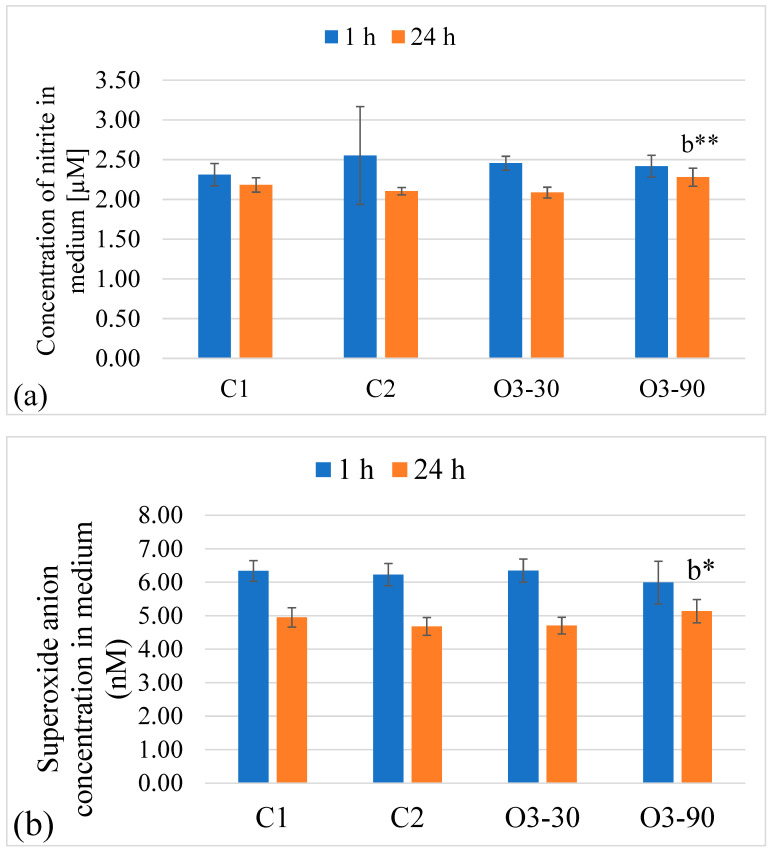
Nitrite (**a**) and superoxide (**b**) production in monocytes following ozone exposure. (**a**) Nitrite concentrations measured by the Griess assay and (**b**) superoxide generation determined by the NBT assay after 1 h and 24 h incubation. Bars represent mean ± SD (n = 3 biological replicates, each measured in duplicate). Statistical analysis was performed using two-way ANOVA with Tukey’s multiple comparison test. Significant differences were observed only for C2 (**b**) and are indicated as * *p* ≤ 0.05, ** *p* ≤ 0.01 (Tukey’s test). No statistically significant differences were detected for C1 (**a**). Visual overlap of SD error bars does not preclude statistically significant differences.

Ozone exposure induced only subtle changes in monocyte nitrite production. At 1 h, no significant differences were observed between treatment groups, while by 24 h a modest overall effect emerged, driven by slightly higher nitrite levels in the high ozone group compared with the air and moderate ozone conditions. Time-dependent reductions were present in both ozone-treated groups. Mean ± SD values and significant post hoc comparisons are shown in Figure 5a, and full ANOVA statistics are presented in Table 4.

Superoxide generation followed a similar pattern. No treatment effects were detected at 1 h, and although a statistically significant overall effect appeared at 24 h, post hoc testing did not reveal pairwise differences between groups. A consistent decline between 1 h and 24 h was observed in most conditions. Mean ± SD values and significance indicators are shown in Figure 5b, while ANOVA model outputs are summarised in Table 4.

## 4. Discussion

This study evaluated the effects of two O_3_ exposure concentrations regimens on porcine neutrophils and monocytes under in vitro conditions. The applied concentrations, 30 µg/mL and 90 µg/mL, corresponded to moderate and high levels of oxidative stress, respectively. The lower dose falls within the range commonly used in clinical MBO in humans and experimental studies, mainly conducted on human-derived cells. In the latter, ozone is observed to exert hormetic and immunomodulatory effects without inducing marked cytotoxicity. In contrast, 90 µg/mL exceeds the widely accepted therapeutic window and, in our model served as a representation of excessive oxidative burden, while remaining non-lethal for most leukocytes. To capture different phases of the cellular response, two time points were analysed: 1 h, reflecting acute oxidative and degranulation events, and 24 h, allowing assessment of delayed effects such as loss of viability, exhaustion, or metabolic adaptation. Functional analyses included RONS generation, release of granular enzymes, cell viability, and monocyte-to-macrophage differentiation, enabling an integrated evaluation of both early and long-term effects of exposure [71,72,73,74].

Growing evidence indicates that ozone can be regarded as an endogenous component of the ROS family, participating in both physiological and pathological redox signalling [75]. From this perspective, its effects are not limited to the generation of secondary mediators but may also involve direct interactions with cellular structures. The reproducible ozone concentrations achieved in the culture medium in our study suggest that the observed responses were not solely the result of rapid chemical reactions with medium components, but also reflected direct cellular effects dependent on concentration, exposure time, and the antioxidant potential of the cells. Against this background, our findings clearly distinguish between adaptive and cytotoxic responses.

### 4.1. Cytotoxic Effects

The observed decrease in neutrophil viability after exposure to O_3_ reflects its clear, dose-dependent cytotoxic potential. At 30 µg/mL, only a mild and non-significant reduction in metabolic activity was detected, suggesting a tolerable level of oxidative stress. In contrast, exposure to 90 µg/mL reduced viability to below 40% of the control value, indicating that the cytotoxic threshold had been exceeded. These results are consistent with previous reports that excessive ozone exposure disrupts mitochondrial function and membrane integrity through uncontrolled RONS generation [29,76].

Our findings confirm that a moderate dose (30 µg/mL) induces only minimal and reversible effects, whereas 90 µg/mL results in substantial loss of viability. Similar dose-dependent responses have been described in other cellular systems, where ozone was shown to induce ROS generation, mitochondrial dysfunction, and apoptosis in epithelial cells, as well as to activate the intrinsic apoptotic pathway (cytochrome c, caspases) in tumour cells [77]. Reviews emphasise that oxidative damage to lipids, proteins, and DNA constitutes a central mechanism of ozone-induced cytotoxicity [78]. In immune cells, ozone exposure has been associated with increased mitochondrial ROS production and inflammasome activation [79]. Taken together, this supports our interpretation that neutrophils can tolerate moderate oxidative stress, whereas exposure to 90 µg/mL overwhelms their defence capacity, leading to massive cell death.

It should be noted that the stronger cellular effects observed at 90 µg/mL likely reflect the integrated impact of both higher concentration and longer exposure. Given ozone’s short lifetime in solution, the two regimens correspond to distinct cumulative oxidative loads rather than strictly concentration-driven responses. Moreover, cells exposed to airflow alone also exhibited a slight reduction in viability compared with untreated controls. This phenomenon is consistent with earlier reports that even clean air in air–liquid interface models can induce mild mechanical stress and transient changes in cell condition. However, this effect was marginal compared with the pronounced cytotoxicity observed with ozone, confirming that the main responses were attributable to O_3_ [80].

Morphological analysis of monocytes differentiating into macrophages further highlighted the dual character of ozone. At 30 µg/mL, cells displayed enhanced spreading and pseudopodia formation—features consistent with activation and differentiation. This suggests that moderate oxidative stress may promote cytoskeletal remodelling (e.g., via Rho/Rac GTPases and actin reorganisation) and support the transition from monocytes to macrophages. Previous studies reported that ROS regulate the actin cytoskeleton by modulating polymerisation and depolymerisation, thereby influencing cell migration and morphology [81,82].

In contrast, exposure to 90 µg/mL resulted in impaired adhesion and a rounded cell shape, indicative of cytotoxic stress, disrupted differentiation, and impaired maturation. High ROS levels have been shown to cause cytoplasmic stiffening and reduced deformability, which negatively affect cell morphology and function [83]. Similarly, cytoskeletal rearrangements in macrophages, including reorganisation of actin filaments, have been described as biphasic depending on the intensity of inflammatory stimuli [84].

Although morphological observations provide valuable insights, their qualitative nature limits the strength of conclusions and warrants cautious interpretation. Future studies incorporating macrophage surface markers (e.g., CD68, CD80, CD163) and cytoskeletal imaging (e.g., phalloidin staining for F-actin, confocal microscopy) could provide quantitative evidence to substantiate these findings.

### 4.2. RONS Generation

The observed changes in RONS production indicate that ozone exerts dose- and time-dependent effects on the oxidative activity of neutrophils, whereas monocytes were less reactive under the tested conditions. In neutrophils, nitrite levels followed a biphasic pattern: the slight decrease after exposure to air and 30 µg/mL O_3_ may reflect early NO consumption by reactive oxygen species or transient inhibition of nitric oxide synthase, while the marked increase after exposure to 90 µg/mL suggests iNOS activation or enhanced conversion of NO precursors under oxidative stress [29,85,86,87,88,89]. In addition, the reduced nitrite production observed at 30 µg/mL may also be related to the increase in arginase activity demonstrated in this study (Figure 1b). Since arginase competes with nitric oxide synthase for the common substrate L-arginine, enhanced arginase activity may limit substrate availability for iNOS, thereby suppressing NO generation and, consequently, nitrite accumulation [90].

The profile of superoxide anion further supports this interpretation: the high ozone dose significantly increased O_2_•^−^ generation at 1 h, consistent with NADPH oxidase activation and an oxidative burst [91,92]. At 24 h the levels declined but remained slightly elevated, suggesting partial resolution or feedback inhibition. The absence of significant changes after short exposure or in the air control group further confirms the existence of an oxidative activation threshold [93,94,95,96].

In contrast to neutrophils, monocytes displayed a suppressed oxidative response, with stable nitrite levels across all conditions and time points. This suggests that ozone did not substantially activate the NO pathway in these cells, which may be explained by their lower basal capacity to generate ROS, stricter regulation of redox signalling, or a potential tolerance mechanism related to inducible iNOS expression [97,98,99,100,101,102].

Taken together, these results demonstrate that ozone modulates RONS production in neutrophils in a concentration-dependent manner. At 30 µg/mL it inhibited nitrite and superoxide generation, suggesting that mild oxidative stress may suppress excessive neutrophil activation. At 90 µg/mL it induced a pronounced oxidative burst and RONS increase, followed by a decline consistent with exhaustion or cytotoxicity. This biphasic pattern reflects the concept of hormesis, in which low doses of an oxidative stimulus promote regulation, whereas higher doses become harmful [103,104]. Clinically, this is relevant since neutrophil-derived RONS are key mediators of tissue injury in chronic inflammatory disorders, including osteoarthritis. Controlled ozone exposure may therefore represent an immunomodulatory strategy to fine-tune neutrophil activity, reducing harmful oxidative responses while preserving functional capacity [105].

### 4.3. Enzymatic Neutrophil Activity

Our data demonstrates that ozone modulates neutrophil degranulation in a dose- and time-dependent manner, with distinct effects on different granule enzymes. MPO and elastase, both markers of azurophilic granule release, showed the most consistent induction following high-dose exposure (90 µg/mL). A marked increase was observed at 1 h post-treatment, and for MPO this effect persisted up to 24 h, indicating sustained oxidative activation rather than a transient response. In contrast, low-dose ozone (30 µg/mL) produced only modest or transient changes, suggesting that azurophilic granule release is triggered once a threshold of oxidative stress is exceeded [106,107]. The early suppression of MPO and elastase release at 30 µg/mL, followed by strong induction at 90 µg/mL, reflects a biphasic pattern that parallels our RONS data, where mild oxidative stress attenuates activation while higher doses drive acute degranulation and oxidative burst. At 24 h, elastase levels normalised, whereas MPO release remained elevated, suggesting that neutrophils sustain certain oxidative pathways over time while limiting proteolytic activity. MPO is recognised as a key indicator of oxidative neutrophil activation with roles in both host defence and the pathogenesis of chronic inflammation [108]. Neutrophil elastase contributes to extracellular-matrix degradation and mediator modulation, and excessive activity is linked to tissue injury [109]. Our observations align with reports that ozone exposure can enhance neutrophil degranulation and increase their pro-inflammatory potential [110].

The release of ALP, a marker of specific granules, exhibited a distinct profile compared to azurophilic enzymes. High-dose ozone enhanced ALP release at early time points, but activity declined after 24 h, consistent with partial resolution, degradation, or reduced secretion as cells underwent stress. Interestingly, at 30 µg/mL, ALP activity decreased significantly after 24 h, suggesting that mild oxidative stress may suppress specific granule release and thereby dampen pro-inflammatory signalling [111]. This is in keeping with the hierarchical model of neutrophil exocytosis, in which secretory vesicles and specific granules respond earlier and are more susceptible to redox modulation than azurophilic granules [112,113].

Arginase activity was robustly induced in a dose-dependent fashion, with the highest activity detected after exposure to 90 µg/mL ozone. As a key regulator of L-arginine metabolism, arginase competes with nitric oxide synthase for substrate availability, potentially reducing nitric oxide production while promoting ornithine, proline, and polyamine synthesis. Such metabolic reprogramming may contribute to tissue repair and immunoregulatory responses. Even low-dose ozone modestly increased arginase activity, consistent with the role of sublethal oxidative stress as a signalling stimulus [67,114,115]. The modest elevation observed in the air control group further suggests that mechanical gas exposure alone can impose mild stress on neutrophils, though this effect was small compared to the ozone-specific induction.

In summary, neutrophils are highly sensitive to ozone exposure and undergo complex, dose-dependent remodelling of enzymatic activity. Excessive stimulation at high ozone concentrations promotes cytotoxic and pro-inflammatory degranulation, characterised by sustained MPO release and transient elastase secretion. In contrast, controlled exposure to lower concentrations appears to modulate neutrophil function by suppressing the release of azurophilic and specific granule contents while enhancing arginase activity. This dual response highlights ozone’s potential to regulate neutrophil behaviour, while simultaneously emphasising the importance of defining a precise therapeutic window to avoid harmful effects.

Overall, the present results suggest that low ozone exposure (30 µg/mL) does not merely activate neutrophils but may instead promote a more controlled redox state. At this dose, ozone was associated with limited RONS generation, absence of marked azurophilic degranulation, and enhanced arginase activity, which could potentially influence nitric oxide metabolism through competition for L-arginine. Such changes may reflect a shift towards a more regulated oxidative and enzymatic profile. Importantly, this response differed clearly from the cytotoxic and pro-inflammatory effects observed at 90 µg/mL ozone.

### 4.4. Study Limitations

Several limitations of this study should be acknowledged. First, neutrophil enzymatic activity was assessed using extracellular release and colorimetric assays, which provide indirect measures and do not fully capture intracellular dynamics or the contribution of distinct granule subsets. Second, while MPO, elastase, ALP, and arginase are representative mediators, they reflect only a fraction of the complex neutrophil degranulation repertoire. Third, the observed increase in enzyme release and arginase activity cannot be clearly distinguished between active immunomodulation and passive leakage due to cytotoxic damage, particularly at the higher ozone dose. Finally, the experiments were performed under simplified in vitro conditions, which do not fully reproduce the in vivo microenvironment where interactions with other immune and stromal cells, as well as systemic antioxidant defences, are likely to modulate neutrophil responses. A further limitation relates to the transient nature of ozone in liquid phase. Because achieving target concentrations required continuous bubbling for different durations, concentration and exposure time could not be separated experimentally. The present data therefore describe cellular responses to a cumulative oxidative dose; future studies incorporating real-time ozone decay measurements or kinetic modelling would allow a more precise estimation of effective exposure.

## 5. Conclusions

The experimental design applied in this study enabled a comprehensive assessment of ozone effects on innate immune cells and provided new insights into leukocyte tolerance thresholds and the immunomodulatory properties of O_3_. The findings clearly demonstrate that both dose and exposure time are critical determinants of cellular responses. The lower concentration (30 µg/mL for 10 min) exerted a mild, potentially modulatory effect, whereas the higher dose (90 µg/mL for 30 min) triggered pronounced oxidative stress and cytotoxicity. Neutrophils responded more strongly than monocytes, underscoring their role as early oxidative sensors. The enzymatic profile revealed preferential activation of azurophilic granules and a marked increase in arginase activity, suggesting that part of the response shifted toward pathways associated with the resolution of the inflammatory process. At the same time, the results highlight the dual nature of ozone: when applied at appropriate doses, it may exert regulatory effects, but excessive exposure becomes detrimental. Placing these findings within the One Health framework emphasises the shared oxidative and immunological mechanisms across humans and animals, as well as the relevance of environmental ozone exposure. Understanding cross-species responses to ozone is essential for the safe biomedical use of this gas and for limiting its immunotoxic and ecological risks. The present work provides a foundation for future studies aimed at optimising ozone dose, exposure time and mechanistic understanding in a translational context.

## Figures and Tables

**Table 1:** Statistical outcomes for neutrophil metabolic activity (MTT) and arginase activity following ozone exposure.

Parameter	MTT Assay	Arginase Activity
One-way ANOVA (F, df)	F(3,12) = 174.67	F(3,12) = 75.58
*p*-value	≤0.0001	<0.0001
Effect size (η^2^)	0.98	0.95
Observed power (1 − β)	1.00	1.00

**Table 2:** Statistical outcomes for neutrophil MPO, ALP, and elastase release at 1 h and 24 h.

Parameter	MPO 1 h	MPO 24 h	ALP 1 h	ALP 24 h	Elastase 1 h	Elastase 24 h
ANOVA (F, df)	F(3,60) = 45.29	F(3,60) = 21.76	F(3,60) = 23.36	F(3,60) = 12.69	F(3,60) = 11.48	F(3,60) = 2.16
*p*-value	<0.0001	<0.0001	<0.0001	<0.0001	<0.0001	n.s.
η^2^ (effect size)	0.694	0.521	0.540	0.390	0.365	n.s.
Observed power	1.00	1.00	1.00	0.99	0.98	—

**Table 3:** Statistical outcomes for nitrite and superoxide production at 1 h and 24 h following ozone exposure.

Parameter	Nitrite 1 h	Nitrite 24 h	Superoxide 1 h	Superoxide 24 h
ANOVA (F, df)	F(3,60) = 203.01	F(3,60) = 7.24	F(3,60) = 172.79	F(3,60) = 1.96
*p*-value	<0.0001	0.00032	<0.0001	n.s.
η^2^ (effect size)	0.91	0.27	0.90	n.s.
Observed power	1.00	0.97	1.00	—

**Table 4:** Statistical outcomes for monocyte nitrite and superoxide production at 1 h and 24 h following ozone exposure.

Parameter	Nitrite 1 h	Nitrite 24 h	Superoxide 1 h	Superoxide 24 h
ANOVA (F, df)	F(3,20) = 0.55	F(3,20) = 5.91	F(3,20) = 0.87	F(3,20) = 3.29
*p*-value	n.s.	0.0047	n.s.	0.0417
η^2^ (effect size)	0.08	0.47	0.12	0.33
Observed power	—	0.88	—	0.58

## Data Availability

The data presented in this study are available on request from the corresponding author due to privacy.

## Supplementary Materials

The following supporting information can be downloaded at: https://www.mdpi.com/article/10.3390/ani16020193/s1, Supplementary Note S1: Spectrophotometric Determination of Ozone Concentration in Cell Suspensions. This section describes the methodology used to quantify dissolved ozone in culture medium following 10- and 30-min ozone exposures, including the iodometric assay, calibration curve preparation, and calculation of cumulative oxidative dose; Figure S1: Calibration curve for the determination of ozone concentration in culture medium using the iodometric method. Standard iodine (I_2_) solutions were prepared in phosphate buffer (pH 6.0), and their absorbance was measured at 352 nm. Linear regression equation: y = 0.0374x (R^2^ > 0.99), where x is the ozone concentration (µg/mL) and y is the absorbance. References [116,117,118] are cited in the Supplementary Materials.

## Abbreviations

The following abbreviations are used in this manuscript:

4-HNE	4-hydroxynonenal
ALP	Alkaline Phosphatase
ANOVA	Analysis of Variance
ATP	Adenosine Triphosphate
CT	Computed Tomography
DMEM	Dulbecco’s Modified Eagle Medium
FBS	Fetal Bovine Serum
GM-CSF	Granulocyte-Macrophage Colony-Stimulating Factor
HO-1	Heme Oxygenase-1
I_2_	Molecular Iodine
iNOS	Inducible Nitric Oxide Synthase
Keap1	Kelch-like ECH-associated protein 1
LOPs	Lipid Ozonation Products
MBOAH	Major Blood Ozonation
M-CSF	Macrophage Colony-Stimulating Factor
MPO	Myeloperoxidase
MRI	Magnetic Resonance Imaging
MTT	[3-(4,5-dimethylthiazol-2-yl)-2,5-diphenyl tetrazolium bromide]
n.s.	not significant
NADH	Nicotinamide Adenine Dinucleotide
NO	Nitric Oxide
NOS	Nitric Oxide Synthase
Nrf2	Nuclear factor erythroid 2-related factor 2
O_2_	Molecular Oxygen
O_3_	Ozone
PBS	Phosphate-Buffered Saline
RONS	Reactive Oxygen and Nitrogen Species
ROS	Reactive Oxygen Species
SD	Standard Deviation
