# Supplementary material for: Mild Ozone-Induced Oxidative Stress Modulates the Activity and Viability of Porcine Neutrophils and Monocytes
Source: Animals (Basel). 2026 Jan 8;16(2):193. doi: 10.3390/ani16020193 (PMC12838241; doi:10.3390/ani16020193)
Supplement: Supplementary file 1 [file animals-16-00193-s001.zip › animals-4050560-supplementary.pdf]

Supplementary Note S1

S.1.1. Spectrophotometric Determination of Ozone Concentration in Cell Suspensions

Ozone exposure was performed using a MALATEC (Legnica, Poland) O_3 generator (nominal O_3 output: 400 mg/h), which produces ozone via corona discharge. Only a small fraction of the generated ozone dissolves in aqueous media, and the effective concentration depends on temperature, generator output and gas delivery characteristics. At the experimentally verified gas flow rate of 2 L/min (120 L/h), the ozone concentration in the O_2/O_3 mixture was approximately 3.3 mg/L.

To quantify dissolved ozone in the culture medium following 10- and 30-minute exposures at room temperature (≈25 °C), we used an iodometric method. A calibration curve was generated from standard molecular iodine (I_2) solutions prepared in phosphate buffer (pH 6.0), and absorbance was measured at 352 nm according to established protocols [1–3]. Each calibration point represents the mean value ± SD of three independent measurements, ensuring reproducibility and minimising measurement variability.

The calibration curve showed strong linearity ($R^2 > 0.99$), described by the Equation (S1):

$$y = 0.0374x, \quad \text{(S1)}$$

where y is the absorbance at 352 nm, and x is the iodine concentration in μg/mL (Figure S1). Experimental samples (ozonated culture medium) were mixed 1:1 with 2 % KI, incubated for 30 minutes in the dark at 4 °C and measured at 352 nm. Dissolved ozone concentrations were calculated from the equivalent I_2 concentration using the molar mass of I_2 (253.8 g/mol) [2,3]. Based on these measurements, ozone concentrations in the medium reached approximately 30 μg/mL after 10 minutes and 90 μg/mL after 30 minutes at 25 °C. When normalised to the 20 mL suspension volume, the cumulative gas-phase ozone delivered to the samples corresponded to approximately 33 mg·min/L (10 min) and 100 mg·min/L (30 min), whereas only ~0.9 % of this ozone was recovered in dissolved form. These results indicate that the two exposure regimens represent distinct cumulative oxidative doses, rather than fully independent concentration variables, and this should be considered when interpreting the cellular responses.

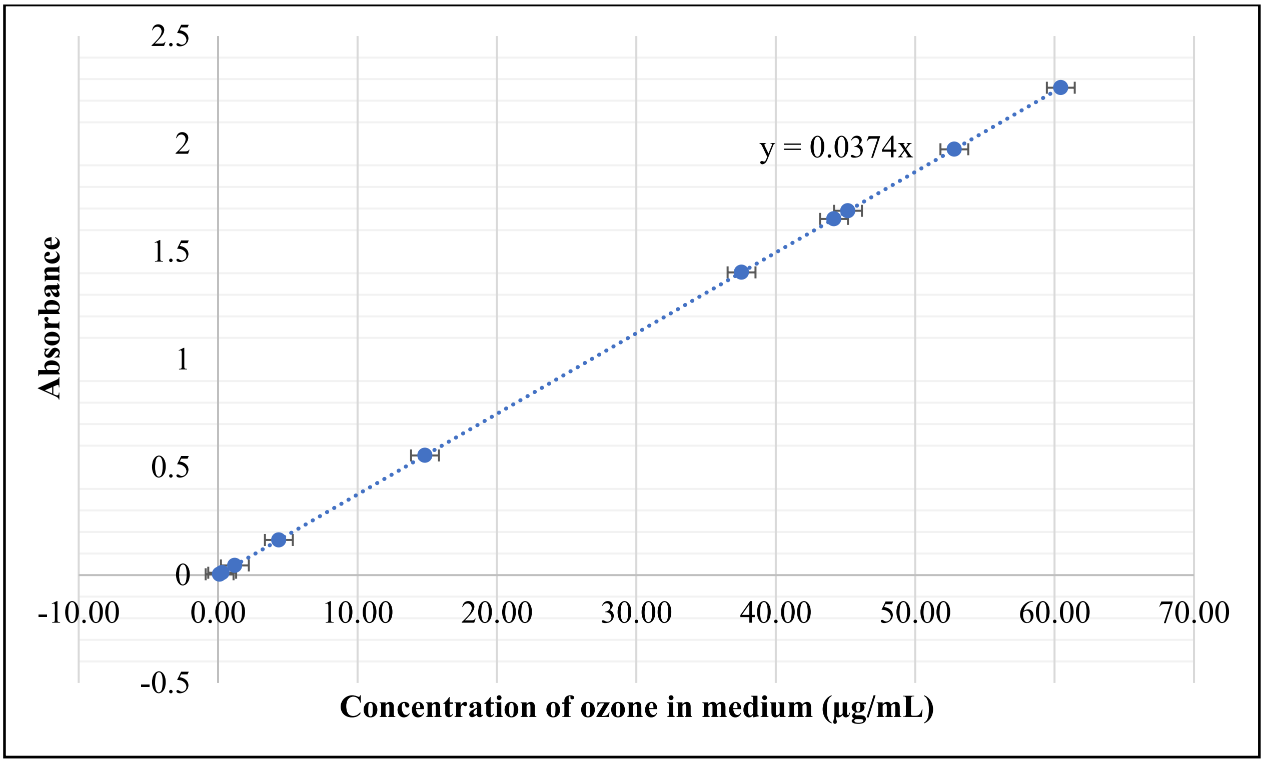

Figure S1. Calibration curve for the determination of ozone concentration in culture medium using the iodometric method. Standard iodine (I_2) solutions were prepared in phosphate buffer (pH 6.0), and their absorbance was measured at 352 nm. Linear regression equation: y = 0.0374x ($R^2 > 0.99$), where x is the ozone concentration (µg/mL) and y is the absorbance.

1. Shechter, H. Spectrophotometric Method for Determination of Ozone in Aqueous Solutions. *Water Research* **1973**, *7*, 729–739, doi:10.1016/0043-1354(73)90089-4.
2. Afkhami, A.; Madrakian, T.; Zarei, A.R. Spectrophotometric Determination of Periodate, Iodate and Bromate Mixtures Based on Their Reaction with Iodide. *ANAL. SCI.* **2001**, *17*, 1199–1202, doi:10.2116/analsci.17.1199.
3. Santos, L.M.C. dos; Silva, E.S. da; Oliveira, F.O.; Rodrigues, L. de A.P.; Neves, P.R.F.; Meira, C.S.; Moreira, G.A.F.; Lobato, G.M.; Nascimento, C.; Gerhardt, M.; et al. Ozonized Water in Microbial Control: Analysis of the Stability, In Vitro Biocidal Potential, and Cytotoxicity. *Biology* **2021**, *10*, doi:10.3390/biology10060525.
